# Impact of gratitude on posttraumatic growth in patients with coronary stent implantation: the mediating role of resilience and perceived social support

**DOI:** 10.3389/fpubh.2025.1513861

**Published:** 2025-01-23

**Authors:** Shanyan Lei, Yujie Zhang, Fang Yang

**Affiliations:** ^1^Department of Rehabilitation Medicine, The First Affiliated Hospital, Zhejiang University School of Medicine, Hangzhou, China; ^2^School of Humanities and Management, Zhejiang Chinese Medical University, Hangzhou, China

**Keywords:** coronary stent implantation, gratitude, posttraumatic growth, model, mediating effect

## Abstract

**Introduction:**

This study focused on investigating the extent of posttraumatic growth (PTG) and explored how resilience and perceived social support (PSS) mediate the relationship between gratitude and PTG among patients with a history of coronary stent implantation.

**Methods:**

A total of 242 patients with coronary stent implantation completed the Posttraumatic Growth Inventory, the Gratitude Questionnaire-Six Item Form, the Resilience Scale, and the Perceived Social Support Scale. We used structural equation modeling to evaluate the mediating effects of resilience and PSS on gratitude and PTG.

**Results:**

The mean score for posttraumatic growth was 55.54 (standard deviation = 15.01). Gratitude had a direct positive effect on PTG in patients with coronary stents (*β* = 0.126) and an indirect positive effect through the mediating effects of resilience and PSS (*β* = 0.105 and 0.081, respectively). Furthermore, resilience and PSS acted through serial multiple mediation effects in the relationship between gratitude and PTG.

**Discussion:**

Gratitude positively affected PTG directly and indirectly through the single-and chain-mediating effects of resilience and PSS in patients with coronary stent implantation. These findings offer compelling evidence of the key interrelating mechanisms among protective factors that contribute to PTG. Therefore, accounting for the predictive influence of gratitude, resilience, and PSS in PTG when developing relevant intervention strategies may help improve patients’ quality of life.

## 1 Introduction

### 1.1 Concept and impact of posttraumatic growth in patients with coronary stent implantation

Coronary heart disease (CHD) involves the narrowing of coronary arteries caused by fatty deposits, reducing blood flow to the heart and potentially leading to angina, shortness of breath, or heart attacks (1). To address this condition, percutaneous coronary intervention (PCI) has become the primary clinical treatment (2). PCI is a minimally invasive procedure designed to restore blood flow to the heart using stents or drug balloon implantations (3). According to registry data released in a report on the development of healthcare quality and technical capacity in China by China’s National Health Commission, the total number of registered PCI cases in mainland China in 2023 was 1,636,055, reflecting a 26.44% increase compared to in 2022 (4). The number of coronary interventions has proliferated following the COVID-19 pandemic. However, despite its clinical benefits, many patients with PCI experience postoperative anxiety, dysphoria, depression, and other adverse emotions. These negative emotions interact with the pathological changes caused by the disease, resulting in a vicious cycle (5). Therefore, addressing the psychological state of patients with PCI is crucial.

Researchers have recently shown increased interest in posttraumatic growth (PTG), a positive transformation from grappling with highly challenging life crises. PTG presents in various forms, including heightened gratefulness for life, deeper interpersonal relationships, enhanced personal strength, and redefined priorities (6). As an essential concept in positive psychology, PTG has demonstrated numerous benefits in individuals with chronic illnesses or medical events, such as cancer, multiple sclerosis, rheumatic diseases, and spinal cord injury (7). One study found that patients with CHD with a high level of PTG tend to be more attentive to their physical and mental health and have a better prognosis (8). Similarly, Affleck et al. (9) found that patients who perceived positive psychological changes after their first myocardial infarction (MI) were less likely to experience a second MI 8 years later. Therefore, it is important to focus on PTG in patients treated with PCI.

### 1.2 Theoretical framework and model construction

Recently, a growing body of research has highlighted the profound influence of positive psychological factors on PTG. Traits such as optimism, hope, and social support have significantly enhanced PTG by fostering adaptive coping mechanisms and promoting meaning reconstruction in adversity (10–13). A core concept from a positive psychological perspective is gratitude. Gratitude is an emotional trait that encompasses both the emotions of gratitude and the disposition to be grateful. The emotion of gratitude pertains to an individual’s expression of appreciation for assistance received. Similarly, the disposition of gratitude denotes a general trend to recognize and appreciate generosity and help from others, experience the emotion of gratitude, and respond accordingly (14–18). Therefore, exploring PTG from the perspective of gratitude in patients with PCI represents a novel and valuable research avenue.

Gratitude broaden-and-build theory, rooted in Fredrickson’s broaden-and build theory of positive emotions, is one of the most successful theoretical models explaining how gratitude enhances individuals’ social adaptability and promotes personal growth (19). According to gratitude broaden-and-build theory, gratitude, as a positive emotion, initially broadens individuals’ immediate thoughts and actions, encouraging them to adopt more expansive and flexible cognitive and behavioral strategies. Through this broadening effect, gratitude further helps individuals build enduring personal resources, including psychological resources (such as resilience) and social resources (such as perceived social support). The accumulation of these resources, in turn, enhances individuals’ sense of well-being and personal growth, forming an upward spiral process: gratitude continuously broadens thought and action, while the resources constructed reinforce individuals’ gratitude experiences, ultimately fostering long-term well-being and personal development (20). A core proposition of this theory is that “gratitude builds personal resources.” As such, the higher an individual’s level of gratitude, the more comprehensive their resources become, which in turn leads to greater well-being and personal growth. Through this process of broadening and building, gratitude helps individuals accumulate long-term adaptive resources that are especially crucial in coping with adversity, enhancing psychological resilience, and fostering personal growth. Thus, gratitude not only has short-term emotional benefits but also promotes psychological growth and recovery following trauma by building resources (21).

A revised model of PTG proposed by Tedeschi et al. (22) offers a complementary perspective by emphasizing how individuals achieve positive psychological change through the process of meaning reconstruction after trauma. According to this model, PTG arises from a deliberate cognitive process of reinterpreting and finding meaning in traumatic experiences. This process is facilitated by the interplay of internal psychological resources, such as resilience, and external social resources, such as perceived social support (PSS). These resources help individuals navigate the cognitive dissonance caused by trauma, enabling them to reinterpret their experiences and foster growth in domains such as personal strength, interpersonal relationships, and a renewed appreciation for life.

Therefore, this study focuses on how gratitude influences PTG in patients with coronary stent implantation. By integrating the above two theoretical models, this study selects key variables to construct patients’ psychological capital (resilience) and social resources (PSS), ultimately facilitating their growth.

### 1.3 Gratitude and PTG

Empirical support for the relationship between gratitude and posttraumatic growth (PTG) is substantial. For instance, Fredrickson (19, 20) demonstrated that positive emotions such as gratitude can counterbalance the negative emotional responses following trauma, helping individuals experience positive psychological outcomes during the recovery process. Several cross-sectional studies have demonstrated that gratitude is positively correlated with PTG scores and moderates the adverse impact of various risk and traumatic factors on PTG. The stronger the gratitude of the individual, the easier it is to promote their PTG (23–26). However, studies explicitly exploring the relationship between gratitude and PTG in patients undergoing PCI or those with CHD still need to be included. This represents a significant research gap, highlighting the need for further investigation in these specific populations. Therefore, we propose the following hypothesis:

*Hypothesis 1*: Gratitude directly affects PTG in patients with coronary stent implantation.

### 1.4 Gratitude, resilience, and PTG

Resilience is a pivotal concept in positive psychology. The American Psychological Association defines resilience as an individual’s ability to successfully adapt when confronted with hardship, distress, risks, and other significant negative life events (27). Recent research has shown a robust link between resilience and PTG. Wang et al. (28) found that psychiatric nurses with strong resilience exhibited higher PTG levels than those with poor resilience. Similarly, Yi et al.’s (29) study found that resilience and rumination were positively correlated with PTG in 540 front-line healthcare workers treating patients diagnosed with COVID-19. Moreover, several studies have indicated a significant relationship between gratitude and resilience. Alkozei et al. (30) found that elevated levels of trait gratitude were linked to the cultivation of personal resources, including resilience. At the same time, studies have found that gratitude has a positive correlation with resilience (31, 32). A study on Iranian veterans with post-traumatic stress disorder showed that gratitude indirectly influenced PTG through the mediating role of ego resilience, highlighting the transformative power of resilience in translating gratitude into psychological growth (33). Therefore, we also propose the following hypothesis:

*Hypothesis 2*: Gratitude indirectly affects PTG levels through resilience in patients with coronary stent implantation, as resilience mediates the relationship between gratitude and PTG.

### 1.5 Gratitude, PSS, and PTG

Perceived social support (34) encompasses an individual’s assessment of social support, focusing on their subjective feelings and experiences related to that support (35). Research has shown that higher PSS levels are linked to greater PTG. Feng et al. (36) demonstrated a positive correlation between PSS and PTG in Chinese patients with gynecological cancer. Wu et al. (37) showed that the direct impact of PSS on PTG could be quantified as 0.32. The psychological mechanism is that PSS provides emotional and instrumental support, enabling individuals to cope with significant life changes and reframe traumatic experiences positively, thereby promoting PTG. Furthermore, a number of cross-sectional studies have suggested an association between gratitude and PSS. For example, social support has been recognized as playing a mediating role between gratitude and depressive symptoms; in other words, more grateful individuals tend to report greater social support and lower levels of depression (38). Similarly, social support has been reported as a mediator in the relationship between gratitude and cardiovascular reactivity (39). Therefore, we proposed the following hypothesis:

*Hypothesis 3*: Gratitude indirectly affects PTG in patients with coronary stent implantation through PSS, as PSS mediates the relationship between gratitude and PTG.

### 1.6 Resilience and PSS

According to the conservation of resources (COR) theory, individuals have a fundamental tendency to conserve, protect, and acquire resources (40). Both potential resource loss threats and actual resource losses can lead to significant stress and tension. COR theory further posits that in stressful situations, individuals mobilize various personal resources to cope with stress and utilize existing resources to acquire new ones, thereby minimizing net resource loss.

Patients with coronary stent implantation experience immense psychological stress owing to heart attacks and surgical treatments. Based on understandings drawn from the COR theory, patients with PCI may draw on multiple personal resources to manage their stress and acquire new resources to reduce net resource loss. As a crucial personal resource, resilience would enable such individuals to adapt quickly, recover from adversity, and achieve growth in the face of disease-related challenges. Resilient patients are also more likely to seek and acquire new resources, including external resources such as social support, to further mitigate resource loss.

Resilient patients demonstrate a higher capacity to mobilize and utilize external resources, such as PSS, which helps them navigate the psychological and physical stress of surgery (41). By strengthening their ability to perceive and use social support, resilient patients are better equipped to manage the stress associated with medical procedures, thereby enhancing their recovery outcomes. These conclusions are consistent with findings from a study by Ong et al. (42), which demonstrated that resilience is closely associated with an increased capacity to perceive and utilize social support. Similarly, Sexton et al. (43) demonstrated that resilient caregivers were more likely to employ positive coping strategies, express their concerns, and seek compassionate listeners. Therefore, we propose the following hypothesis:

*Hypothesis 4*: There is a serial multiple mediation effect of gratitude on PTG through resilience and then through PSS.

### 1.7 Research purpose

**Figure 1:**
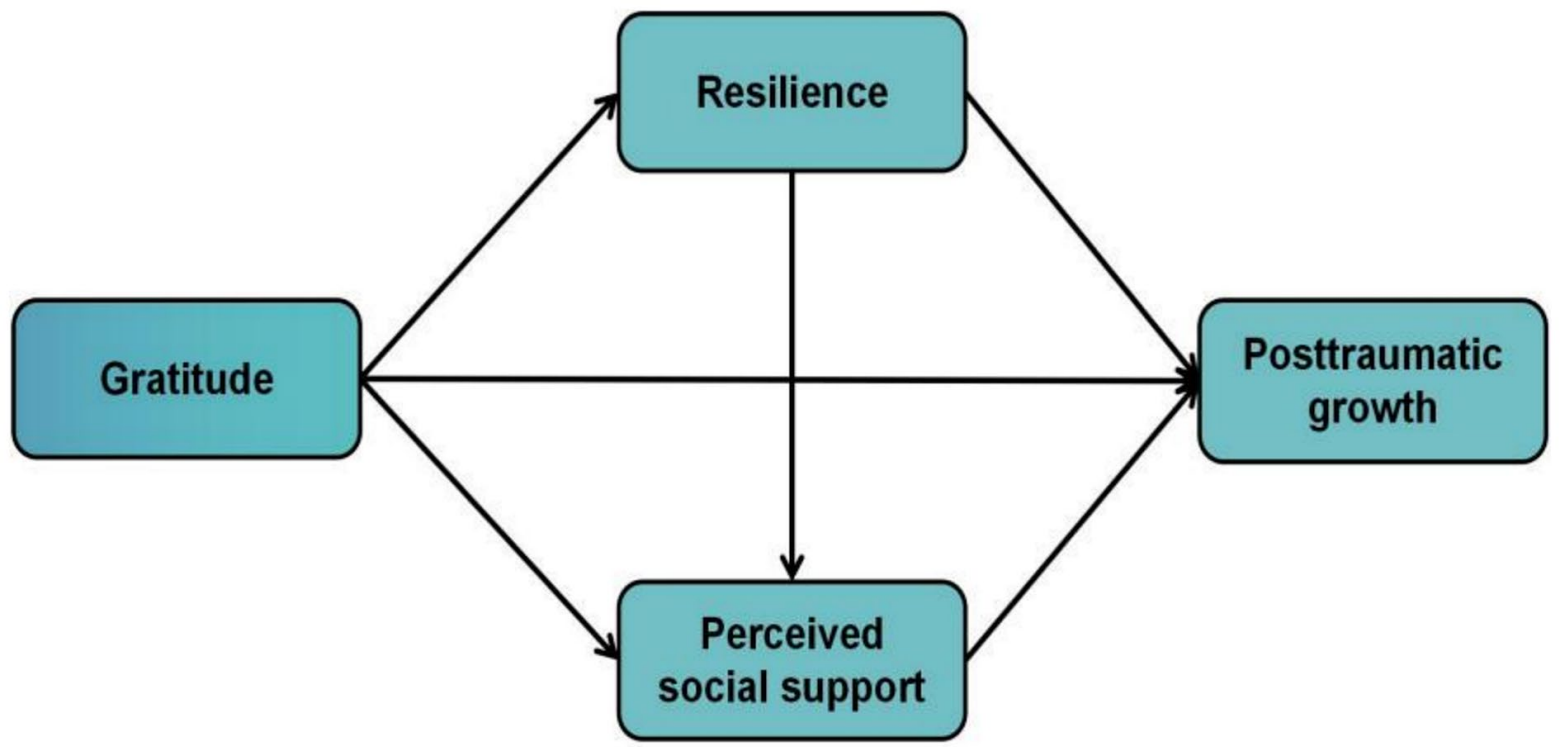
Hypothesis model.

Given that gratitude positively impacts PTG and adaptation to adversity in traumatized individuals (16–19), exploring patients’ gratitude’s effects and psychological mechanisms on PTG provides a theoretical basis for targeted gratitude interventions (44). It is well-known that a collectivist culture prevails in Chinese society (45), and research into the mechanisms of gratitude on PTG in China may expand cultural comprehension of PTG. So far, however, there has been little discussion about PTG in patients with coronary stent implantation. To address this knowledge gap, the current study aimed to investigate the extent of PTG and explore how resilience and PSS mediate the relationship between gratitude and PTG among patients with a history of coronary stent implantation. This study contributes to both theory and practice by exploring the applicability of the broaden-and-build theory, the revised PTG model, and the COR theory in Chinese patients with coronary stent implantation. It also examines gratitude as a potential entry point for developing interventions to help patients cope with traumatic stress, reduce negative emotions, improve postoperative growth, and inspire future research on gratitude and PTG across diverse populations. Therefore, based on theoretical and literature research, the hypothesis model is shown in Figure 1.

## 2 Methods

### 2.1 Setting and participants

To conduct this cross-sectional study, a convenience sampling method was chosen due to its feasibility and accessibility. Three general hospitals in Hangzhou, the capital city of Zhejiang Province, China, were selected based on their size, diversity of patient populations, and the hospitals’ willingness to collaborate. Eligible patients were identified after obtaining their written informed consent, which also authorized their treating physicians to share anonymized medical records with the research team. This process followed ethical guidelines approved by the hospital’s ethics committee, ensuring patient privacy and voluntary participation. Patients who met the following inclusion criteria were approached by trained research staff during their hospital stay shortly before discharge: (1) diagnosed with coronary artery disease by coronary angiography; (2) treated with PCI for the first time; (3) stabilized after surgery and able to be discharged; (4) no cognitive impairment; (5) consented to participate in the study. The recruitment process involved face-to-face interactions where the study purpose, procedures, and voluntary nature of participation were clearly explained, and written informed consent was obtained from each participant. Patients who had a traumatic experience within 6 months or other serious diseases, or who had verbal communication barriers, were excluded from the study. Questionnaires were administered to patients who had previously undergone coronary stent implantation and met the inclusion criteria. Of the 260 questionnaires distributed, 242 were completed and returned, yielding an effective response rate of 93.08%. Given the small proportion and randomness of the missing data, multiple imputation methods were employed to handle missing values. Specifically, five imputations were performed using predictive mean matching, and the results were pooled for the final analysis.

As presented in Tables 1, 2, the mean (SD) age of the 242 patients was 64.88 (11.36) years, ranging from 30 to 88 years. Within the full sample, 77.3% were men, 86.4% were married, 52.5% were retired, 54.5% were living with children, 45.5% were living without children (departed from home), 87.2% were non-religious, 67.4% understood their health condition completely, 75.6% reported monthly income more than or equal to 3,000 RMB, and 58.3% were living in a city, while 41.7% were living in the countryside. Approximately 46.7% of the participants reported having one coronary stent, and 13.2% had more than three stents. Moreover, approximately 77.7% of the participants said they would never return to work.

**Table 1:** Participants’ characteristics (*n* = 242).

Participant	Group	*n* (%)	Participant	Group	*n* (%)
Age (years)	<50	27 (11.1)	Work status	Unemployed	55 (22.7)
	50–59	45 (18.6)		Sick leave	7 (2.9)
	60–69	87 (36.0)		Retired	127 (52.5)
	70–79	57 (23.6)		Part-time	6 (2.5)
	≥80	26 (10.7)		Full-time	47 (19.4)
Gender	Male	187 (77.3)	Family origin	City	141 (58.3)
	Female	55 (22.7)		Countryside	101 (41.7)
Religion	Yes	31 (12.8)	Family status	Children departed	110 (45.5)
	No	211 (87.2)		With children	132 (54.5)
Monthly income (RMB)	<1,000	8 (3.3)	Marital status	Unmarried	6 (2.5)
	1,000–2,000	12 (5.0)		Married	209 (86.4)
	2,001–3,000	68 (28.1)		Divorced	2 (0.8)
	>3,000	154 (63.6)		Widowed	25 (10.3)
Payment of expenses	Medical insurance	228 (94.2)	Disease awareness	Completely unaware	7 (2.9)
	Self-funded	14 (5.8)		Partially informed	72 (29.8)
Number of stents	1	113 (46.7)		Completely aware	163 (67.3)
	2	62 (25.6)	Education level	Illiterate	37 (15.3)
	3	35 (14.5)		Elementary school	76 (31.4)
	>3	32 (13.2)		Middle school	75 (31.0)
Return to work	Yes	54 (22.3)		High school	24 (9.9)
	No	188 (77.7)		College or above	30 (12.4)

**Table 2:** Descriptive data and correlation analyses between variables.

Variables	Mean (SD)	Gratitude	Equanimity	Self-reliance	Ceaseless self-improvement	Meaningfulness	Home support	Out-of-home support	Appreciation for life	Personal strength	New possibilities	Compared to others	Spiritual change
Gratitude	28.90 ± 3.73	1.000											
Equanimity	31.80 ± 5.00	0.159^*^	1.000										
Self-reliance	31.32 ± 5.25	0.175^**^	0.647^**^	1.000									
Ceaseless self-improvement	36.76 ± 5.59	0.177^**^	0.567^**^	0.710^**^	1.000								
Meaningfulness	31.67 ± 5.51	0.250^**^	0.533^**^	0.683^**^	0.621^**^	1.000							
Home support	23.90 ± 3.66	0.292^**^	0.200^**^	0.275^**^	0.199^**^	0.285^**^	1.000						
Out-of-home support	41.64 ± 8.83	0.330^**^	0.189^**^	0.296^**^	0.220^**^	0.445^**^	0.567^**^	1.000					
Appreciation for life	18.11 ± 5.13	0.333^**^	0.349^**^	0.422^**^	0.346^**^	0.383^**^	0.354^**^	0.361^**^	1.000				
Personal strength	8.86 ± 2.59	0.182^**^	0.298^**^	0.410^**^	0.322^**^	0.423^**^	0.303^**^	0.348^**^	0.730^**^	1.000			
New possibilities	10.14 ± 3.97	0.246^**^	0.251^**^	0.296^**^	0.258^**^	0.346^**^	0.295^**^	0.359^**^	0.726^**^	0.652^**^	1.000		
Compared to others	9.16 ± 2.62	0.388^**^	0.181^**^	0.204^**^	0.127^*^	0.229^**^	0.375^**^	0.280^**^	0.581^**^	0.402^**^	0.456^**^	1.000	
Spiritual change	9.35 ± 3.55	0.213^**^	0.271^**^	0.433^**^	0.404^**^	0.417^**^	0.230^**^	0.330^**^	0.684^**^	0.619^**^	0.569^**^	0.405^**^	1.000

^**^p < 0.01, ^*^P < 0.05. SD, standard deviation.

### 2.2 Ethical considerations

This study was performed in compliance with the principles of the Declaration of Helsinki. Prior to commencing the study, ethical approval was obtained from the Research Ethics Committee of the First Affiliated Hospital, College of Medicine, Zhejiang University (ethical approval number: 2018462). After obtaining informed consent from patients, the questionnaires were completed anonymously with the help of an investigator.

### 2.3 Instruments

Demographic characteristics were collected via a general information questionnaire, including age, gender, religion, monthly income, payment of medical expenses, number of stents, work status, family status, marital status, disease awareness, and education level.

#### 2.3.1 Gratitude questionnaire-six item scale

Gratitude was assessed among participants using the Gratitude Questionnaire-Six Item Scale (GQ-6) (21), which has been translated into Chinese by Fang (46). This questionnaire is a self-report and unidimensional instrument designed to evaluate individuals’ experiences and manifestations of gratitude and appreciation in their daily lives, along with their perceptions of the benefits received from others. The questionnaire comprises six items, and each item is rated on a seven-point Likert scale (1 = strongly disagree, 7 = strongly agree). Items 3 and 6 were reverse coded. The questionnaire comprises six items, each rated on a seven-point Likert scale (1 = strongly disagree, 7 = strongly agree), with items 3 and 6 being reverse coded. Response options range from strongly disagree, disagree, somewhat disagree, neutral, somewhat agree, agree, to strongly agree. An example item is: “I think there’s a lot to be thankful for in life.” The confirmatory factor analysis (CFA) indices were as follows: ratio of chi-squared statistic to the degrees of freedom *χ^2^*/df = 2.50, comparative fit index (CFI) = 0.95, Tucker–Lewis Index (TLI) = 0.93, and root mean square error of approximation (RMSEA) = 0.05. The Chinese version of the GQ-6 has demonstrated favorable reliability, and the Cronbach’s *α* in the current sample was 0.687.

#### 2.3.2 Resilience scale

Resilience was assessed using the Resilience Scale (RS) (47), which has been translated into Chinese by Yang et al. (48). It contains 25 items across four dimensions: equanimity, ceaseless self-improvement, meaningfulness, and self-reliance. Each item is graded on a Likert scale. Total scale scores range from 25 to 175, with a score under 125 signifying a low level of resilience, scores ranging from 125 to 145 signifying a moderate level of resilience, and scores above 145 representing a high level of resilience. Response options range from strongly disagree, disagree, somewhat disagree, neutral, somewhat agree, agree, to strongly agree. An example item is: “I can depend on myself more than anyone else.” The psychometric properties of the Chinese version of the RS have been well-validated. The CFA indices were as follows: *χ^2^*/df = 2.77, CFI = 0.91, TLI = 0.90, and RMSEA = 0.07. The Cronbach’s *α* in the current sample was 0.875, and the Cronbach’s α for the subscales ranged from 0.580 to 0.694.

#### 2.3.3 Perceived social support scale

The Perceived Social Support Scale (PSSS) (49), as translated by Jiang (50), was used to assess individual perceptions of social support. The PSSS consists of 12 items in two dimensions: home support and out-of-home support. Each item is graded on a Likert scale, with possible scores ranging from 12 to 84. Response options range from strongly disagree, disagree, somewhat disagree, neutral, somewhat agree, agree, to strongly agree. An example item is: “I can count on my friends in times of trouble.” The psychometric properties of this scale have been validated in Chinese populations (Cronbach’s *α* in this total scale, 0.960; Cronbach’s α of the home support subscale, 0.926; Cronbach’s α of the out-of-home support subscale, 0.936). The PSSS generally shows a good fit in different populations. The CFA indices were as follows: *χ^2^*/df = 2.45, CFI = 0.92, TLI = 0.91, and RMSEA = 0.06. In this study, the Cronbach’s *α* in the total scale was 0.864, while the home support and out-of-home support subscales had Cronbach’s α values of 0.680 and 0.873, respectively.

#### 2.3.4 Posttraumatic growth inventory

The PTG was assessed using the PTG Inventory (PTGI), as translated into Chinese by Wang et al. (51). The PTGI comprises 20 items categorized into five domains: personal strength (three items), relation to others (three items), new possibilities (four items), spiritual change (four items), and appreciation of life (six items). A Likert scale is used to evaluate each item, with potential scores ranging from 0 to 100. Response options range from not at all, a little, somewhat, moderately, quite substantially, to extremely. An example item is: “I have a greater appreciation for the value of my life.” The properties of the PTGI have been validated in Chinese populations. The PTGI has also demonstrated a good fit across studies, with CFA indices as follows: *χ^2^*/df = 2.38, CFI = 0.93, TLI = 0.92, and RMSEA = 0.06. In this study, the Cronbach’s *α* in the current sample was 0.887, and the Cronbach’s α for the subscales ranged from 0.655 to 0.788.

### 2.4 Data analysis

The data were entered into Microsoft Excel (Microsoft Corp., Redmond, WA, United States) and analyzed using the statistical analysis software SPSS 27.0 and Amos 26.0 (IBM Corp., Armonk, NY, United States). Descriptive statistics are presented as means and standard deviations (SDs) for continuous variables and as counts and percentages for categorical variables. Pearson product–moment correlation coefficients were computed to evaluate the associations between gratitude, resilience, PSS, and PTG. Statistical significance was set at 0.05. A structural equation model (SEM) was used to examine the mediating effects of resilience and PSS on gratitude and PTG. To assess whether the indicators accurately represented the latent variables, measurement models were initially estimated using a series of CFA models. Additionally, we assessed the model’s goodness of fit using the following indices: the chi-square statistic (*χ*^2^/df < 3), goodness of fit index (GFI; ≥0.90), adjusted goodness of fit index (AGFI; ≥0.90), normed fit index (NFI; ≥0.90), robust fit index (RFI; ≥0.90), incremental fit index (IFI; ≥0.90), TLI (≥0.90), CFI (≥0.90), and RMSEA (<0.05) (52). The mediation effects of resilience and PSS were evaluated using the Bootstrapping method with 5,000 resamples, as implemented in Amos 26.0. Bootstrapping provides bias-corrected confidence intervals (CI) for indirect effects, making it a robust and reliable method for testing mediation effects without assuming normality. Indirect effects were considered significant if the 95% CI did not include zero. This method was used to test the following mediation pathways:

(1) Gratitude → Resilience → PTG
(2) Gratitude → PSS → PTG
(3) Gratitude → Resilience → PSS → PTG

## 3 Results

### 3.1 Correlation analyses between variables

The results of the correlation matrix indicated that gratitude was positively associated with all dimensions of resilience, PSS, and PTG. All dimensions of resilience were positively correlated with PSS and PTG. PSS in each dimension was also positively associated with all dimensions of PTG. The correlations were generally moderate (|*r*| = 0.13 to 0.73, all *p* < 0.05). The results are summarized in Table 2.

### 3.2 Mediating effect of resilience and PSS on gratitude and PTG

An SEM was constructed according to the above results to further investigate the relationship between gratitude, resilience, PSS, and PTG in patients with coronary stent implantation. Table 3 shows the model fit indices: *χ*^2^/df = 1.240, GFI = 0.960, AGFI = 0.937, NFI = 0.921, RFI = 0.963, IFI = 0.993, TLI = 0.989, CFI = 0.992, and RMSEA = 0.032. These values indicate that the model has a good fit. The ratio of *χ*^2^/df (1.240) is well below the commonly accepted threshold of three, suggesting a good fit. GFI (0.960) and AGFI (0.937) exceed the recommended cutoffs of 0.90, further supporting the model’s fit. NFI (0.921) and RFI (0.963) are above 0.90, indicating a good incremental fit. IFI (0.993), TLI (0.989), and CFI (0.992) all exceed the common threshold of 0.90, with IFI and CFI both closer to one, suggesting an excellent fit. Finally, RMSEA (0.032) is well below the threshold of 0.05, indicating a close fit. These values collectively suggest that the model fits the data well. The model has 49 degrees of freedom, and no correlations were established between the error terms, further supporting the adequacy of the model fit. Additionally, no significant changes were observed in the model estimates when monthly income and education level were introduced as control variables. Therefore, we infer that there were no confounding effects.

**Table 3:** The model fitting parameters.

Fit Index	CMIN/DF	GFI	NFI	CFI	IFI	PNFI	PCFI	RMSEA
Reference value	<3	>0.9	>0.9	>0.9	>0.9	>0.6	>0.6	<0.08
Correction value	2.267	0.926	0.925	0.956	0.957	0.687	0.710	0.0073

CMIN/DF, chi-square statistic/degrees of freedom; GFI, goodness of fit index; NFI, normed fit index; CFI, comparative fit index; IFI, incremental fit index; PNFI, parsimonious NFI; PCFI, parsimonious PCFI; RMSEA, root mean square error of approximation.

**Figure 2:**
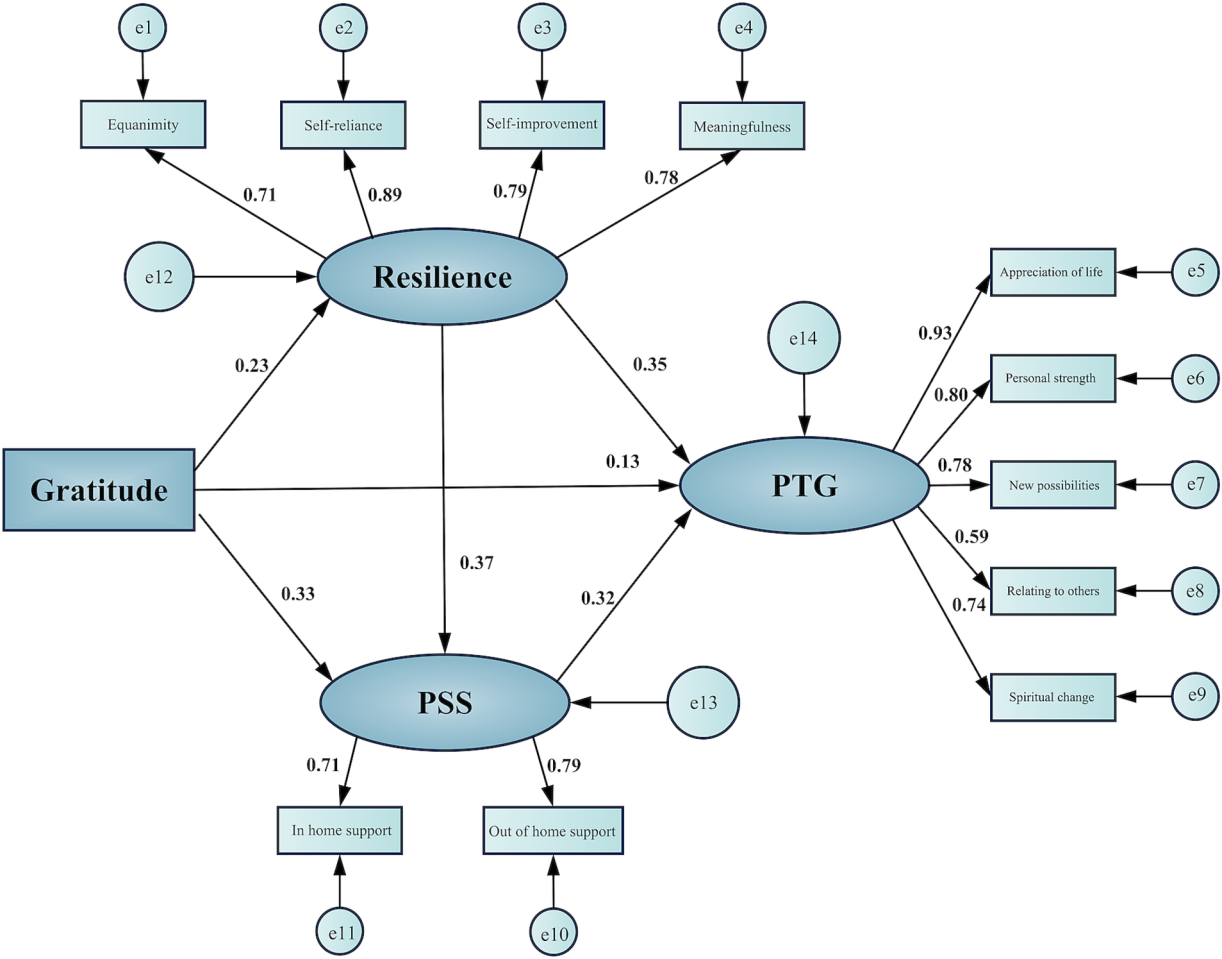
Structural equation model: resilience and perceived social support mediating the association between gratitude and posttraumatic growth. PSS, perceived social support; PTG, posttraumatic growth.

As revealed in Figure 2 and in Table 4, the results of SEM showed that gratitude significantly and positively influenced PTG (*β* = 0.126, *p* = 0.049); Hypothesis 1 was confirmed. Gratitude significantly and positively influenced resilience (*β* = 0.229, *p* < 0.001) and resilience significantly and positively influenced PTG (*β* = 0.352, *p* < 0.001). Gratitude significantly and positively influenced PSS (*β* = 0.329, *p* < 0.001) and PSS significantly and positively influenced PTG (*β* = 0.318, *p* < 0.001). Resilience significantly and positively influenced PSS (*β* = 0.366, *p* < 0.001).

**Table 4:** Path coefficients between variables.

Dependent variable	Independent variable	SE	CR	*P*	*β*
Resilience	Gratitude	0.065	3.35	<0.001	0.229
PSS	Gratitude	0.134	4.618	<0.001	0.329
PSS	Resilience	0.158	4.55	<0.001	0.366
PTG	Gratitude	0.082	1.966	0.049	0.126
PTG	PSS	0.061	3.525	<0.001	0.318
PTG	Resilience	0.098	4.795	<0.001	0.352

PSS, perceived social support; PTG, posttraumatic growth.

As shown in Table 5, the individual indirect effects were as follows: (1) The “Gratitude → Resilience → PTG” pathway had an effect size of 0.081, accounting for 23.89% of the total effect, 95% CI [0.045, 0.132], *p* < 0.001. (2) The “Gratitude → PSS → PTG” pathway had an effect size of 0.105, accounting for 30.97% of the total effect, 95% CI [0.062, 0.160], *p* < 0.001. (3) The “Gratitude → Resilience → PSS → PTG” pathway had an effect size of 0.027, representing 7.96% of the total effect, 95% CI [0.010, 0.048], *p* = 0.004. The direct effect of gratitude on PTG was 0.126, which accounted for 37.17% of the total effect. All these effect sizes were accompanied by bias-corrected 95% CIs that did not include zero, which confirms the significance of both the direct and indirect effects. These results support the partial mediation effects outlined in Hypotheses 2, 3, and 4. In summary, gratitude in patients with coronary stent implantation directly affected PTG and indirectly affected PTG through the mediating effects of resilience and PSS. Resilience and PSS exhibited serial multiple mediation effects in the relationship between gratitude and PTG.

**Table 5:** Percentage of path coefficients.

Effect	Path	*β*	Bias-corrected 95% CI	Proportion
Total effect	Gratitude → PTG	0.339	[0.178,0.500]	100%
Direct effect	Gratitude → PTG	0.126	[0.035,0.287]	37.17%
Indirect effect		0.213	[0.184,0.242]	62.83%
Indirect effect 1	Gratitude → Resilience → PTG	0.081	[0.069,0.093]	23.89%
Indirect effect 2	Gratitude → PSS → PTG	0.105	[0.089,0.121]	30.97%
Indirect effect 3	Gratitude → Resilience → PSS → PTG	0.027	[0.026,0.028]	7.96%

CI, confidence interval; PSS, perceived social support; PTG, posttraumatic growth.

## 4 Discussion

This study explored the extent of PTG, focusing on the potential roles of resilience and PSS in the relationship between gratitude and PTG among patients with coronary stent implantation in China.

Our findings showed that the mean (SD) PTGI score among patients with coronary stent implantation in Hangzhou was 55.54 ± 15.01. This score was higher than the PTG levels reported in Canada (50.30 ± 27.20) among CAD outpatients (12) and in Israel (41.3 ± 27.3) among patients following MI and coronary artery bypass grafting (CABG) (53) and lower than in Australia (70.14 ± 21.11) among patients post-surgical CABG (54). In Canada, PTG varied significantly by ethnicity, with Chinese participants associated with the lowest scores, possibly owing to the small sample size of the study. The Israeli study measured PTG within 6 months of the cardiac event and thus focused on more acute cases, which may have contributed to lower PTG in that study. The higher PTG observed in Australian patients following CABG may have been influenced by various factors, including differences in patient experience and recovery. Our findings closely align with those of a study (55) from the United States and the United Kingdom involving patients with heart disease (55.85 ± 24.19), suggesting that PTG levels among coronary stent patients in China are moderate and comparable internationally, with variations likely influenced by differences in illness severity, treatment type, and cultural factors. When comparing our results to other types of patients, we found that the mean PTG score among patients with colorectal cancer varied, ranging between 58.05 and 76.78 (56), whereas that among patients with acquired brain injury was 47.95 ± 23.75 (57). The mean score for PTG among stroke survivors was 51.53 ± 26.25 (58). These results indicate that Chinese patients with coronary stent implantation have moderately low PTG levels. Discrepancies observed in other studies may be attributed to various other confounding factors.

Our results indicate a direct positive effect of gratitude on PTG among patients with coronary stent implantation, supporting Hypothesis 1, consistent with previous research (24, 59, 60). This may be because grateful individuals experience greater happiness, optimism, positive affect, pride, and hope (26, 61, 62). After experiencing trauma, such individuals tend to identify positive resources in their lives and develop positive perceptions of both their environment and themselves (63). Moreover, individuals who practice gratitude often reframe traumatic events or situations through a cognitive lens, aiming to derive meaning and achieve a better understanding (64, 65). Kim and Bae (66) showed that an increased sense of gratitude enhances the effect of deliberate rumination on PTG. The influence of gratitude-activated deliberate rumination on PTG may serve as a buffer against intrusive stressor-related thoughts (67, 68). Additionally, according to broaden-and-build theory related to positive emotions (69), gratitude can broaden thought-action repertoires, assist patients in recovering from negative emotional experiences, and promote behaviors that significantly aid their recovery, such as quitting smoking and maintaining a balanced diet.

Consistent with Hypothesis 2, our findings revealed that resilience played a mediating role in the relationship between gratitude and PTG among patients with coronary stent implantation. Specifically, grateful patients were more likely to have better resilience, resulting in greater PTG. Gratitude is an experience of thankfulness, encompassing the appreciation of positive experiences in daily life (70). Cultivating positive emotions associated with gratitude to manage negative emotions can initiate an upward spiral toward enhanced well-being, thereby fostering resilience against adverse outcomes (69, 71). Additionally, fostering a grateful disposition broadens cognitive and creative capacities, enabling individuals to consider various actions to express their gratitude (20). Therefore, with these skills, patients with coronary stent implantation become more resilient, reflective, socially integrated, and healthy, thus contributing to PTG (72). This finding aligns with previous studies showing that those who frequently experience and express gratitude often show higher levels of resilience (18, 32, 73). Eyni et al. (33) observed similar results regarding the mediating role of resilience in the relationship between gratitude and PTG among Iranian veterans with posttraumatic stress disorder (75).

Furthermore, in line with Hypothesis 3, PSS partially mediated the relationship between gratitude and PTG in patients with coronary stent implantation. According to previous studies, gratitude alleviates social isolation (74) and strengthens social relationships (75). Patients may be more connected to their family members, friends, or even strangers, facilitating relationship formation and social connections, thereby improving their PSS. Strong PSS can offer the mental resources needed to cope with trauma and effectively alleviate posttraumatic psychological stress. This finding is partially consistent with a study of college students who began their first year in the fall 2020 semester during the COVID-19 pandemic, where gratitude promoted PSS and reduced depressive symptoms, both of which contributed to PTG (76). These findings can be extended to patients undergoing coronary stent implantation.

Consistent with Hypothesis 4, we found that gratitude affected PTG via resilience through PSS. More precisely, grateful patients are inclined to be resilient, which in turn may allow them to experience more PSS, leading to a higher PTG. A positive relationship between resilience and PSS has been reported, such that individuals with more resilience experience greater PSS (42, 77). This finding is inconsistent with a previous study of patients with cancer in Germany, which indicated that individuals with high resilience may rely less on psychosocial support to cope with stressful situations compared to those with low resilience (78). This discrepancy may be attributed to cultural differences. Western culture tends to emphasize individualism and personal freedom (79, 80). Consequently, Western parents often seek to establish a sense of independence for their children at an early age. By contrast, Eastern culture is centered around families, especially in China. Family serves as the primary reference for behavior and decision-making, with all the activities revolving around it, resulting in close-knit family relationships. As a result, resilient Asian patients are more inclined to utilize positive coping strategies, share their concerns with family members or friends, and find sympathetic listeners, resulting in higher PSS (42). The findings of our study align with broaden-and-build theory, which posits that “gratitude expands an individual’s immediate cognitive mindset and builds a range of personal resources, including psychological resilience and social support, ultimately contributing to an individual’s growth and well-being” (70). Thus, resilience and PSS may be important contributors to the relationship between gratitude and PTG in patients with coronary stent implantation.

### 4.1 Limitations

This study has several limitations. First, our findings primarily highlight potential pathways among the studied variables; however, owing to the cross-sectional nature of the data, we cannot confirm the temporal sequence required to establish causality. Second, the use of convenience sampling in this study may have compromised the representativeness of the sample; moreover, geographical constraints could have further impacted sample diversity. Third, our research sample size is relatively modest for the SEM analysis. These factors should be accounted for in future studies, which could also benefit from leveraging open data to address these limitations and overcome information barriers (81).

### 4.2 Advantages

From a theoretical perspective, this study provides robust support for the broaden-and-build theory by extending its application to the context of cardiovascular patients. Specifically, it highlights the essential role of gratitude in fostering PTG, reinforcing the concept that positive emotions broaden cognitive and emotional resources, enhance resilience, and strengthen PSS. Furthermore, this research enriches and refines the theoretical framework of PTG by demonstrating the applicability of the broaden-and-build theory, the revised model of PTG, and the conservation of resources theory within a Chinese cultural context. By examining these psychological mechanisms in patients with coronary stent implantation, the study validates the universality of these theories and uncovers potential cultural nuances, offering a valuable perspective for localized psychological research. Additionally, the interdisciplinary nature of this study, bridging psychology, medicine, and sociology, promotes cross-disciplinary collaboration, paving the way for innovative approaches to patient care and recovery. While most studies explore how PSS influences resilience, our study takes a different approach by examining how resilience affects PSS in patients with PCI. This choice is grounded in the unique psychological challenges faced by patients with PCI, where individual psychological resources, such as resilience, may play a more active role in initiating the acquisition of resources. Resilient individuals are better equipped to perceive and utilize social support, actively seeking and creating supportive networks to cope with the stresses of surgery and recovery. By exploring this less-studied pathway, we offer a complementary perspective to existing literature, enriching our understanding of the dynamic interactions between internal and external resources in stress recovery. Our data analysis further supports this hypothesis, showing that resilience predicts PSS, thus providing empirical evidence for this reverse pathway.

From a practical perspective, the findings emphasize the utility of gratitude-based interventions as a novel and effective approach for enhancing PTG in patients. By incorporating gratitude practices, such as journaling or expressing appreciation, healthcare providers can encourage patients to develop a more positive outlook, strengthen social support networks, and build emotional resilience. These interventions can be seamlessly integrated into existing cardiac rehabilitation programs, offering a more holistic and patient-centered approach to recovery. Moreover, gratitude and PTG, as positive psychological constructs, can improve postoperative adaptation, reduce complications, and promote overall psychological well-being, ultimately enhancing the quality of life for patients with coronary stent implantation. This research provides a practical foundation for designing targeted psychological interventions that support mental and physical recovery by bridging theoretical insights with actionable strategies.

In summary, this study underscores gratitude’s dual value: its theoretical importance in advancing the understanding of PTG and its practical potential in shaping effective, culturally informed strategies for improving the recovery and well-being of patients facing serious health challenges.

## 5 Conclusion

This study adopted a positive psychology approach to provide several key insights into the relationship between gratitude, resilience, PSS, and PTG among patients with coronary stent implantation. First, Chinese patients with coronary stent implantation reported moderate levels of PTG. Second, gratitude was found to have a direct and positive effect on PTG, reinforcing the role of emotional well-being in fostering recovery. Third, resilience and PSS emerged as important mediators, strengthening the connection between gratitude and PTG. These findings suggest that gratitude may enhance PTG by improving resilience and PSS, which are vital in recovery. Finally, the results emphasize the value of promoting gratitude in therapeutic settings to facilitate emotional healing and personal growth, further supporting its role in patient rehabilitation. These results suggest that interventions targeting gratitude could enhance resilience and PSS, thereby promoting PTG in this population. Gratitude interventions, such as keeping a gratitude journal or expressing gratitude, have been successfully introduced as part of chronic illness care in various countries. Additionally, psychological therapies such as cognitive-behavioral therapy or mindfulness-based interventions could help strengthen resilience. Simultaneously, healthcare professionals should provide relevant support and encourage family and friends to offer companionship and care to such patients, fostering a supportive environment that contributes to their psychological well-being and recovery. This integrated approach could lead to more comprehensive strategies in managing and supporting the psychological health of patients with coronary stent implantation, ultimately improving their overall quality of life.

In terms of future research, this study opens up several promising avenues for further exploration. First, conducting larger-scale, multi-center, and prospective studies would be valuable to verify and extend the findings of this research. These studies could provide a broader and more diverse sample, which would help determine the generalizability of the current results across different populations and settings. Additionally, examining the long-term effects of gratitude interventions would offer insights into the sustained benefits of gratitude on PTG and overall well-being among coronary stent implantation patients. Second, future research could focus on developing gratitude-based intervention programs tailored to PCI patients. These interventions could aim to cultivate gratitude as a therapeutic tool to enhance emotional resilience and facilitate PTG. Investigating the effectiveness of such programs would be an important step in advancing gratitude in clinical practice, especially in helping individuals recover and grow from traumatic experiences. Moreover, cross-cultural comparisons could be valuable to understanding how gratitude functions across different cultural contexts, offering a more nuanced understanding of how cultural norms and values influence the relationship between gratitude and PTG. Furthermore, exploring the psychological and physiological mechanisms through which gratitude impacts PTG could provide deeper insights into the underlying processes and help refine intervention strategies.

## Data Availability

The raw data supporting the conclusions of this article will be made available by the authors, without undue reservation.
